# Patterns of Isoform Variation for N Gene Subgenomic mRNAs in Betacoronavirus Transcriptomes

**DOI:** 10.3390/v17010036

**Published:** 2024-12-30

**Authors:** James J. Kelley, Andrey Grigoriev

**Affiliations:** Department of Biology, Center for Computational and Integrative Biology, Rutgers University, Camden, NJ 08102, USA; jimkell@scarletmail.rutgers.edu

**Keywords:** coronaviruses, SARS-CoV-2, transcriptome, N gene, subgenomic mRNA

## Abstract

The nucleocapsid (N) protein is the most expressed protein in later stages of SARS-CoV-2 infection with several important functions. It is translated from a subgenomic mRNA (sgmRNA) formed by template switching during transcription. A recently described translation initiation site (TIS) with a CTG codon in the leader sequence (TIS-L) is out of frame with most structural and accessory genes including the N gene and may act as a translation suppressor. We analyzed multiple sequenced samples infected by SARS-CoV-2 and found that any single variant of this virus produces multiple isoforms of the N sgmRNA. The main isoform starting at TIS-L is out of frame, but two secondary dominant isoforms (present in nearly all samples) were found to restore the reading frame and likely involved in the regulation of N protein production. Analysis of sequenced samples infected by other coronaviruses revealed that such isoforms are also produced in their transcriptomes. In SARS-CoV, they restore the reading frame for a putative TIS (also a CTG codon) in the same relative position as in SARS-CoV-2. Positions of junction breakpoints relative to stem loop 3 in the 5′-UTR suggest similar mechanisms in SARS-CoV, SARS-CoV-2, and OC43, but not in MERS-CoV. These observations may be pertinent for antisense-based antiviral strategies.

## 1. Introduction

Coronaviruses (CoVs) are among the largest plus-strand RNA viruses, with genomes sizing up to ~30,000 nt. General awareness of CoVs has increased due to the recent COVID-19 pandemic and its infectious agent, SARS-CoV-2. All CoV genomes contain multiple open reading frames (ORF) named from plain ORF1a, ORF1b, etc. to more descriptive ones such as spike (S) or nucleocapsid (N) genes. ORF1a and ORF1b comprise two-thirds of the genome on the 5′-end and are directly translated from genomic RNA to produce non-structural proteins, including RNA-dependent RNA polymerase (RdRp). Translation of the remaining third of the genome is much more complex and consists of 3 main stages.

**Figure 1:**
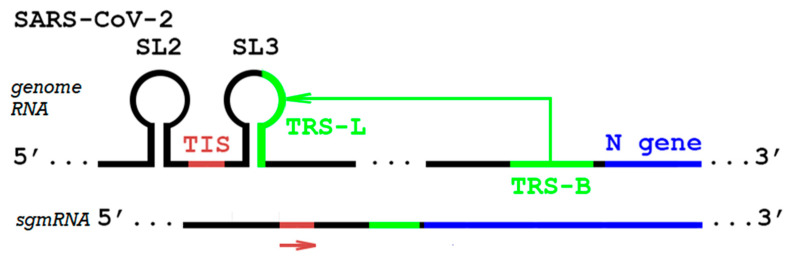
Schematic of the 5′ region and N gene region in SARS-CoV-2 genome (black), connected by template switch (green arrow) when producing sgmRNA template during stage 1. sgmRNA produced during stage 2 is shown at the bottom. Utilization of alternate translation initiation site reported in [4] is indicated by the red arrow; otherwise, the ATG initiation codon at the start of the N gene (blue) is used. See text for further details.

Stage 1, often ignored in papers describing SARS-CoV-2 transcription and its products, involves the synthesis of subgenomic RNAs (sgRNAs) by the RdRp from the plus-strand genomic RNA, followed by stage 2, when these sgRNAs are in turn used as *templates* for transcription of subgenomic mRNAs (*sgmRNA*) [1]. These sgmRNAs are then translated in stage 3 to produce structural and accessory proteins. All sgmRNAs have identical 5′ ends containing a leader sequence found on the 5′ end of the genome. An important landmark for sgmRNAs is a transcription regulatory sequence (TRS); its copy at the 3′ end of the leader is called TRS-leader, or TRS-L. Other TRS copies (often of varying sizes) are also located on the 5′ end of each structural and accessory ORF as TRS-body (TRS-B). During stage 1, the 3′ end of each sgRNA is joined to the leader sequence through discontinuous transcription via template switching by RdRp between TRS-B and TRS-L (Figure 1, items in green). When an infected sample is sequenced, the resulting reads represent a mixture of contiguous genomic RNA and sgmRNA with TRS-based junctions [2]. Each of these junctions has known distinct coordinates and can be detected as deletions after sequencing and aligning the reads to a virus reference genome (and we will refer to them as “deletions” below). Among the structural proteins produced in this manner in SARS-CoV-2, the N protein is the most abundant, responsible for packaging viral RNA, with multiple roles, including those in regulation, sgmRNA transcription, and host innate immune system suppression [3].

Eukaryotic translation can be initiated at translation initiation sites (TIS) by noncanonical start codons CTG, GTG, and TTG along with the canonical ATG codon, with CTG generally the most efficient [5]. In a surprising deviation from the TRS-based model above, a CTG start codon upstream of TRS-L at 59 nt position in the leader sequence was observed in SARS-CoV-2 [4,6] and designated TIS-L (TIS located in the leader; Figure 1, items in red), further noting its role as an upstream ORF (uORF) start codon in many sgmRNAs. These uORF were speculated to function as translational suppressors for most of the respective sgmRNAs [4].

Ribosome-protected mRNA fragment sequencing (RPF-seq) and quantitative profiling of initiating ribosomes sequencing (QTI-seq) were used for quantification of TIS sites. A large fraction of RPF-seq and QTI-seq reads contained TIS-L, and most of these reads were mapped to sgmRNAs [4]. Three uORFs using TIS-L are in-frame with the main gene in their sgmRNA (ORF1a, ORF6, Spike), but others are out of frame (ORFs 3a, E, M, 7a, 7b, 8, and N). These three sgmRNAs and a few out-of-frame ones were analyzed in detail, but the N gene was not among them. Given the importance and abundance of the N gene, we focused our computational investigation on the fine structure of deletions and junctions in sgmRNAs encoding it. Availability of high-coverage transcriptomic data (necessary for observing such fine structure) varies significantly for different CoV genera and is the highest for beta CoVs; it explains our focus on this genus of CoV.

Interestingly, we found multiple isoforms of the N sgmRNA (with different sizes of deletions) in transcriptomes of sequenced SARS-CoV-2 samples and observed that in the main isoform the uORF is out of frame. However, the frame was restored in sequences containing two other deletion isoforms, and these isoforms were omnipresent in different cell culture samples infected by SARS-CoV-2. When we expanded this analysis to other CoVs and observed that such patterns were most similar between SARS-CoV and SARS-CoV-2, with OC43 somewhat similar and with MERS-CoV quite dissimilar, suggesting that they likely reflect the evolutionary trajectories of CoVs.

## 2. Materials and Methods

### Sample Retrieval, Sequence Analysis

Samples were downloaded from the European Nucleotide Archive as fastq files using wget. Adapter sequences (Illumina TruSeq RA3, San Diego, CA, USA) if present at the 3′ ends were removed by cutadapt-1.2.163 [7] using commands such as

cutadapt -a <adapter_sequence> -o output.fastq input_fastq

Fastq files were aligned to the reference sequence for each virus (see below for reference sequences used) with BWA-MEM (bwa-0.7.17) [8] using default parameters, converted to BAM, sorted and indexed using Samtools (v1.3.1) [9]. We used commands such as

samtools view <sample>|grep sequence_of_interest

to locate reads containing each junction and write them to text files. See Supplementary Table S6 for sequences of interest. Sequences start from TIS-L and end at the start codon except for those with start positions at 65 where not enough sequence is present on the left end of the junction to provide a unique match to the left end of the genome. In these cases, 9 bp are added to the left. In RPF samples, mapped reads were short, on average 30 bp, so we had to remove some bases from the right to find matches for most junctions (Supplementary Table S7). For OC43, the sample group we analyzed all had the SNV T59C. We had to adjust our query sequences to include the SNV (Supplementary Table S6). For analysis of SARS-CoV-2 patient samples of the alpha and delta VOCs, query sequences needed to be adjusted for the 1 bp deletion at 28271 bp, and for omicron VOCs, the query sequences needed to be adjusted for the SNV A28271T. Custom Python scripts were used to process the text files. Reads with mapping quality < 30 were excluded from analysis. The remainder of reads supporting each junction were used to determine the percentage of samples containing each junction. For PRJNA661467, the number of mapped reads in each sample was counted using Samtools with the command

samtools view -F 0x40 <sample>|cut -f1|sort -u|wc -l

The counts were used to calculate Reads Per Million (RPM) for each junction.

Repeats were identified using the Repeats search function in SARSNTdb [10].

We checked peptides for exact matches in the human proteome by Blast (https://blast.ncbi.nlm.nih.gov/Blast.cgi), probability to be signal peptides by SignalP (https://services.healthtech.dtu.dk/services/SignalP-5.0/) or to be MHC class I binders by NetMHC (https://services.healthtech.dtu.dk/services/NetMHC-4.0/). Statistical significance of these observations was calculated by the respective servers.

Stem loop coordinates for SARS-CoV-2, SARS-CoV, and MERS-CoV are based on Conde et al. [11] and Yang and Leibowitz [12], for OC43 on Mackeown et al. [13], and for PEDV on Madhugiri et al. [14].

## 3. Results

### 3.1. Variable Lengths of the N Gene sgmRNAs

We set to compare the patterns of biogenesis of the N gene sgmRNAs across CoVs and considered the distances that the RdRp “jumps” during template switches. These distances can be identified as variable sizes of deletions after aligning the reads to virus reference genomes. Our pipeline detected the common patterns and variations in these deletion sizes in four beta CoVs detected in humans (OC43, MERS-CoV, SARS-CoV, and SARS-CoV-2). Available datasets of beta CoV transcriptomes of higher quality are much more numerous compared to other CoVs. For comparison, we also considered the alpha CoV PEDV detected in pigs. In total, we analyzed some 1000 sequencing datasets, including 301 samples of cell lines infected by one of these viruses (Table 1a) and 685 samples from patients infected with SARS-CoV-2, where the infecting VOC was identified in primary papers (Table 1b). In these datasets, we considered deviations from the canonical template switch positions and corresponding deletion sizes (as postulated by TRS-B and TRS-L coordinates) in the respective N gene sgmRNAs (Table 2).

**Figure 2:**
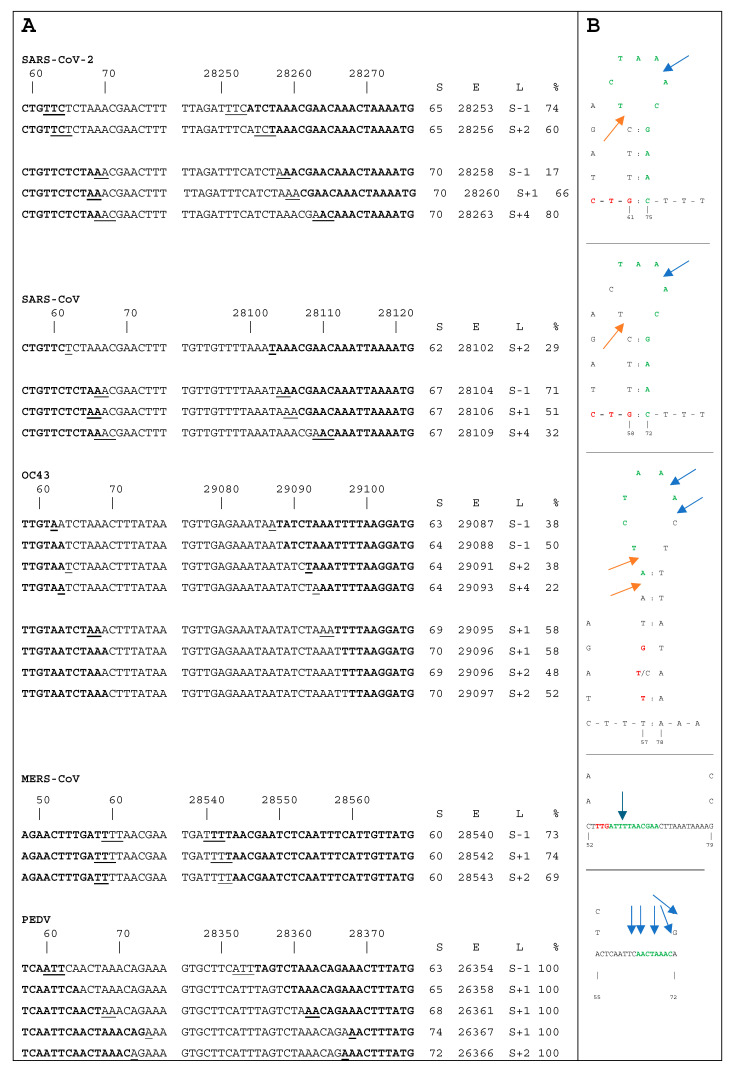
Junction regions in analyzed CoVs (from top to bottom: SARS-CoV-2, SARS-CoV, OC43, MERS-CoV, and PEDV). (**A**) Sequences present in reads, including up- and downstream ends of junctions shown in bold (**left**) with coordinates for each virus genome shown on top. Underlined letters indicate repeated bases on each side of the junction, indicating possible multiple versions of alignments (see Supplementary Figure S1 for further explanations). Alignments between deletions are constructed to show the most common possible breakpoints for each virus, as we assume the template switch landing sites follow the same (virus-specific) localization constraints within the 5′UTR. Tables next to the sequences show start and end coordinates, length in S notation, and sample frequencies (S, E, L, %, respectively). Sample frequencies are the average for all cell lines analyzed for each deletion size for each virus. MERS-CoV averages do not include mouse lung samples. PEDV averages are based on 3 samples. Deletions of size S are not shown as being the most prevalent (Supplementary Figure S1). TRS are shown in bold green, and TIS-L start codons, if present, are in bold red. For MERS-CoV, TTG is part of the TRS. (**B**) Regions around TRS-L in the virus genomes analyzed. Genomes of SARS-CoV-2, SARS-CoV, and OC43 include the SL3 stem loop, shown in full, and the 3′ end of the SL2 depicted as vertical letters. For viruses without the SL3 (MERS-CoV and PEDV), the right end of the SL2 and left end of the SL4 are shown as vertically arranged letters. Arrows indicate the positions of the most common breakpoints.

Interestingly, we observed variable deletion sizes (in addition to the canonical ones) and a variability in lengths of the N gene sgmRNAs within each sample group (Figure 2). We use here a simple mathematical notation to describe the size distribution of these deletions. Let a variable ***S*** represent the canonical deletion size to compare with the size of the deletions observed. We detected size range [***S−1***; ***S+4***] in samples infected with PEDV, OC43, MERS-CoV, SARS-CoV, or SARS-CoV-2.

We summarized our observations in Supplementary Tables S1–S5. Specifically, size ***S*** showed the highest abundance as expected since these reads are translated to produce the very abundant N protein. Deletions of size ***S+1*** were high in samples infected with MERS-CoV, SARS-CoV, or SARS-CoV-2. Size ***S+4*** was high in samples infected with SARS-CoV or SARS-CoV-2 but absent in MERS-CoV and PEDV. Frequencies of the other sizes were much lower in most groups, with ***S+3*** being low or absent. The distribution of isoforms in SARS-CoV-2 patient samples followed that of cell lines infected with SARS-CoV-2, although size ***S+2*** was less frequently observed in the former. We observed a similar behavior with the lower frequencies of isoforms in MERS-CoV mouse lung samples compared to MERS-CoV cell line samples. Comparing different VOCs, we noted that deletions of size ***S-1*** at start position 65 were not found in the two groups of delta samples but were frequent in groups of other VOCs.

### 3.2. 5′ UTR Stem Loop Arrangement and sgmRNA Junction Positions

We analyzed the link between the stem loop arrangement in the 5′ untranslated region (UTR) and the deletions observed. For SARS-CoV-2, SARS-CoV, and OC43 TRS-L occurs on stem loop 3 (SL3), while MERS-CoV does not have a homologous stem loop in this region (Figure 1 and Figure 2). In sequences from the 5′ UTR and from the regions including TRS-B and the start codon of the N gene (Figure 2A), we illustrated common junction points by showing the nucleotides remaining in sgmRNAs in bold and the deleted nucleotides in normal font. In the secondary structures of these 5′ UTR regions (Figure 2B; see Methods for references used for stem loop coordinates), we illustrate junctions by arrows in the secondary structures.

The upstream breakpoints of the most isoforms detected in SARS-CoV-2, SARS-CoV, and OC43 occur on either the 5′ or 3′ side of stem loop SL3. Template switching could occur at these positions as the RdRp complex detects the stem loop, but the isoform variability suggests a possible stochasticity in the junction formation. For SARS-CoV-2, breakpoints for deletions of sizes ***S-1*** (one of two isoforms) and ***S+2*** occur on the left side of the loop while all other breakpoints occur on the right side. For SARS-CoV, only the ***S+2*** breakpoint occurs on the left side of the loop; all others occur on the right side. Notably, for both SARS-CoVs, junctions for ***S+1*** and ***S+4*** occur at one position (right after the loop), suggesting a likely combination of the above stochasticity with structure-related deterministic factors in the template switch process for these junctions.

For OC43, ***S-1***, ***S+2*** (one of two isoforms), and ***S+4*** occur on the left side of the loop, and all others occur on the right side. In MERS-CoV, which does not have the SL3 stem loop homolog following the SL2 in this region, junction positions show no variation based on alignments that minimize the number of breakpoints between deletions; all may start at position 59. PEDV shows yet another pattern of equally frequent deletion isoforms, unlike the beta CoVs.

### 3.3. TIS-L Codons and N Gene sgmRNA Reading Frame Patterns Among Coronaviruses

The non-canonical CTG codon 10 nts upstream of TRS-L was observed in SARS-CoV-2 RPF-seq and QTI-seq reads originating from the TIS-L [4], thus confirming that it is translated. In such reads, deletions of size ***S*** change the reading frame, and a stop codon is produced before the start codon is reached. However, in deletions of size ***S+1*** or ***S+4***, the reading frame is restored for the N gene (Table 3), and the resulting proteins would be the same as the isoforms produced by reads starting from the start codon with an extra seven to eight amino acids added upstream. We performed some standard bioinformatics checks for these upstream peptides and found that they had no exact matches in the human proteome and were unlikely to be signal peptides or MHC class I binders. As for confirmation for these isoforms and peptides with mass spectrometry, we could only see the upstream amino acids in a peptide LFSKRTLKSVWLSLGCMLSALTQYN* already reported [6] as a CTG-driven ORF1a polyprotein isoform. Thus, our statements on translated isoforms below are based on the RPF-seq and QTI-seq data.

A very similar situation is observed in SARS-CoV, which has a CTG codon at 56 bp (in SARS-CoV-2 the TIS-L coordinate is at 59 bp), right between SL2 and SL3 (Figure 2). The CTG codon and TRS-L are in different reading frames for SARS-CoV, and the ***S+1*** and ***S+4*** junctions also restore the reading frame in reads that start at this CTG. Such similarity suggests that in SARS-CoV this codon may also serve as a TIS-L, being a homolog of its SARS-CoV-2 counterpart in terms of sequence identity and identical location between SL2 and SL3.

**Figure 3:**
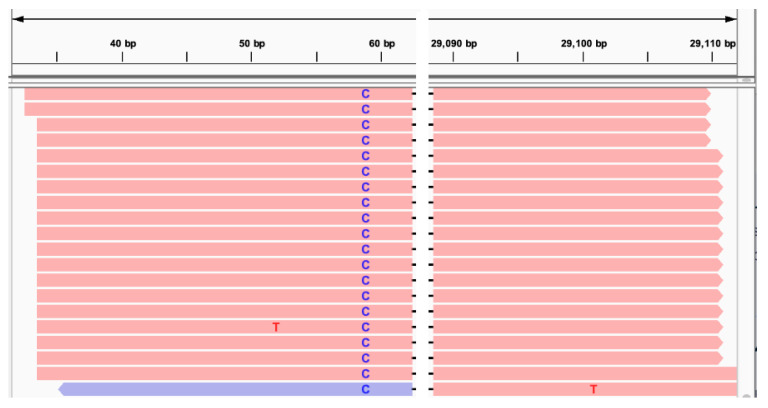
Combined view of two IGV displays with reads of a representative sample infected by OC43 for 5′UTR region (**left**) and the N gene region (**right**). It shows the SNV T59C that would disrupt the start codon at 58 nt.

OC43 has a TTG codon at 58 nt, a less efficient non-canonical start codon than CTG, but it has been observed as a start codon in MHV and IBV [19,20]. This codon is present in the AY391777.1 reference sequence is located on SL3 (or potentially between SL2 and SL3, given some imperfections in SL3 base pairing) and might be a TIS-L homolog. However, it overlaps a single-nucleotide variant (SNV) T59C observed in the samples we analyzed (Figure 3). Such change disrupts this codon and very likely the SL3 (Figure 2). The fact that this codon is not conserved between OC43 samples suggests that it may not be used as a TIS-L or that the SNV may disable TIS-L-related mechanisms.

MERS-CoV also has a TTG codon, closest to being a homolog of TIS-L at location 54 nt (right after the SL2). However, the SL3 is lacking in MERS-CoV, and most of the sgmRNA junctions occur three bases after the codon, unlike the situation in SARS-CoVs (Figure 2).

Finally, we analyzed Calu-3 mRNA-seq and RPF-seq samples from PRJNA661467 infected by SARS-CoV-2 (beta VOC) 1–36 h post-infection (hpi) with 4 RNA-seq samples and 2 RPF-seq samples for each time point. We observed changes in temporal expression of N gene isoforms similar to those of the ORFs analyzed by Kim et al. [4]. All isoforms from Table 3 and Figure 2 (top) were present at 36 hpi in the mRNA-seq and RPF-seq samples (except for ***S-1*** start position 70 in both datasets), confirming they are translated. The missing isoforms in mRNA-seq and RPF-seq are also consistently underrepresented across the samples in Figure 2 and Supplementary Tables S1 and S2.

### 3.4. 1 bp Deletion at 28271 nt Changes TIS-L Reading Frame

While the isoform variation we described above occurs in the transcriptome of CoVs, genomic changes may also affect these patterns. A 1 bp deletion at position 28,271 was reported in B.1.1.7 (alpha) [21] and B.1.167.2 (delta) variant genomes [22]. We considered a supplementary table from an earlier analysis of GISAID consensus sequences [23] and noticed this deletion in 6231 out of 7440 Alpha_B.1.1.7 and 3352 out of 4118 Delta_B.1.617.2 sequences, while not present in significant numbers of sequences in other VOCs. Consistent with this trend, we observed this 1 bp deletion at similarly high frequencies in the patient samples infected by the alpha and delta VOCs from Italy (PRJNA970221) and not for other VOCs. Notably, when this deletion is present, TIS-L and TRS-L are in the same reading frame (although a preceding stop codon is still produced, the ATG of the N gene is in frame and may start translation of the N protein). This might account for the increased expression of N protein observed in alpha samples [24] (and comparably elevated expression at sgmRNA level has been shown in hACE2 mice infected with alpha or delta VOCs [25]). ***S+1*** and ***S+4*** in this case would result in novel proteins (Table 4).

## 4. Discussion

Our analysis of groups of sequenced samples infected by each of the four beta CoVs showed that these viruses have similar isoform distributions of the N sgmRNA yet show some small but likely important differences. Specifically, ***S-1***, ***S+1***, and ***S+2*** isoforms are produced in all four viruses, but the ***S+4*** isoform is only produced in SARS-CoV, SARS-CoV-2, and OC43. We observed ***S-1***, ***S+1***, and ***S+2*** in PEDV (whose patterns differed from beta CoVs), but these results were based on three samples, too few to draw statistically sound conclusions about the prevalence of these isoforms or lack of others.

Kim et al. [4] observed a signal for ORF10 sg mRNA in mRNA-seq and RPF-seq data in late stages (36 hpi) and suggest that ORF10 is translated and may be functional. We found most of the N protein isoforms from Figure 2 in these mRNA-seq and RPF-seq reads; thus, using the same argument, these isoforms should also be translated. ***S+4*** and ***S-1*** have the highest levels at 36 hpi, with ***S+4*** having levels above ORF7b and within an order of magnitude of E and S proteins, which suggests that translation starting at TIS-L may be a significant source of N protein production. TIS-L is in frame for the S protein and appears to enhance production of that protein [4]. Similarly, ***S+1*** and ***S+4*** junctions may be involved in the regulation of N protein production at different stages of infection and for different purposes.

Notably, these translated RPF-seq reads also contain the isoform of size ***S*** with a stop codon (Table 3). To continue synthesis of the N protein with TIS-L from these reads, a +1 frameshift (FS) would be needed. A programmable FS known for CoVs is a –1 FS at the border of ORF1a and ORF1b, and, in a tangential connection to SARS-CoV-2, +1 FS was reported in the N^1^-methylpseudouridylated mRNA sequence of the BNT162b2 vaccine [26]. Is the A-rich sequence context sufficient for enabling +1 FS in this region? Or does it cause sequencing errors leading to the observed isoforms? The latter seems unlikely, as such sequences are found in multiple cell lines infected by both SARS-CoVs in many labs and in patient samples of many VOCs.

The fact that ***S+1*** and ***S+4*** isoforms restore the reading frame and are ubiquitous is striking, but what mechanism enforces that? Selection based on the reading frame is unlikely to operate within a short timeframe of a single infection, yet other isoforms are present in samples much more rarely. Further, the role of the TIS-L-dependent uORF is not well studied, and the function of the upstream peptides in frame with the N protein is unclear (it is neither a signal peptide nor an MHC I binder nor any other decoy similar to host-produced peptides). Earlier work showed that the N-terminal domain uses stacking interactions with a triple adenosine motif to bind the TRS-L in MHV [27]. Could N protein isoforms with these N-terminal peptides attenuate the regulation of their own translation? Interestingly, the arrows of the most frequent junctions in Figure 2 seem to point to the AAA motif. And is the lack of the AAA motif in MERS-CoV (the only TRS without AAA in Table 2) of any consequence for such regulation (or lack thereof) in this virus?

From the arrangement of junction positions conserved between SARS-CoVs (Figure 2), we can see the mechanistic features likely lead to the increased abundance of ***S+1*** and ***S+4***. The ***S+1*** and ***S+4*** isoforms, as well as the arrangement of the SL2, SL3, and TIS-L, suggest that TIS-L functions are very similar in SARS-CoV and in SARS-CoV-2.

The SL2-SL3 pair with a TIS-L between them may serve as a conserved structural beacon in SARS-CoVs, driving their discontinuous transcription via template switching. Further, structural constraints may be imposed by SL3 on only allowing breakpoints on the two sides of the stem loop. The fact that MERS-CoV does not have an SL3 and that the junction positions are constant supports this view. The position of junction breakpoints in SARS-CoV-2, SARS-CoV, and OC43 in SL 3 in the 5′ UTR (Figure 2) indicates the conservation of junction formation in these three viruses. The deletions of size ***S+1*** and ***S+4*** occur on the same side as that of size ***S***, suggesting similar mechanisms in the production of the N protein sgmRNAs. However, ***S+1*** and ***S+4*** do not restore the reading frame in OC43, so their role in this virus may be different. It is worth noting that the TIS-SL3 region is devoid of SNVs in SARS-CoV-2 [23] but not in OC43.

Studies of genome organization may reveal novel targets and inform effective antiviral treatments. Blocking the TIS-L region of SARS-CoV-2 with an antisense oligonucleotide (ASO) [4] has been suggested as a therapeutical approach disrupting virus translational regulation by the TIS-L. If the hypothesized role of ***S+1*** and ***S+4*** in restoring the reading frame in TIS-L-driven translation we describe here is relevant for the N protein production at certain timepoints of the coronavirus lifecycle, ASOs based on these specific junction sequences may enable the development of such treatments for SARS-CoV-2. Virus-specific variations reported in our paper may inform such targeted antiviral strategies for respective CoVs.

Genomic changes to the N gene and its surrounding sequences have been studied in CoVs. Expression level increases in the N protein and ORF9b [24] and the appearance of ORF9c have been reported in SARS-CoV-2 variants [28]. Both ORF9b and ORF9c overlap the N gene in an alternate frame [29], which we also observed as a junction in SARS-CoV-2 samples. It is important to note that the phenomena of isoform length change we described here occur in the transcriptomes, not in the genomes of CoVs. We report changes in the reading frame caused by different isoform lengths in the N sgmRNAs and supported by RPF-seq and QTI-seq reads originating from the TIS-L [4], thus providing further evidence that they can be translated. Such transcriptome changes have not been previously described.

The genomic 1 bp deletion at 28,271 nt causes the start codon to be in frame with TIS-L (Table 4), suggesting that the N protein may be translated even though the start codon is preceded by a stop codon. If two ATGs are present and the first ATG is followed shortly by a terminator codon, reinitiation may occur at the second ATG [30]. Would this still apply when the first codon is CTG instead of ATG? If translation does indeed occur, it may account for some of the increased sgRNA and protein levels in the alpha and delta VOCs. The potential effect of this 1 bp deletion on N protein production suggests that regulation of the N sgmRNA may be more complex than what is currently known.

## 5. Conclusions

Our analysis of hundreds of sequenced samples infected by CoVs found many isoforms of the N sgmRNA. In SARS-CoV-2, the main isoform starting at TIS-L is out of frame, but two ubiquitous secondary isoforms were found to restore the reading frame and are likely involved in the regulation of N protein production. Such isoforms are also produced in other CoVs. As in SARS-CoV-2, in SARS-CoV they restore the reading frame for a putative TIS (also a CTG codon) near the homologous SL3. Positions of junction breakpoints relative to SL3 in the 5′-UTR suggest similar mechanisms in SARS-CoV, SARS-CoV-2, and OC43, but not in MERS-CoV. These observations may be pertinent for antisense-based antiviral strategies.

## Figures and Tables

**Table 1:** (**a**) Projects analyzed, infecting virus, number of samples used in analysis, and cell type infected by virus. ^1^ USA-WA1/2020 isolate; ^2^ Release dates of 09/2020 and 10/2020. ^3^ BetaCoV/Korea/KCDC03/2020; GISAID accession ID: EPI_ISL_407193. (**b**) Analyzed samples from projects of SARS-CoV-2 patients infected with VOCs as identified by the papers in the Reference column.

(**a**)				
**Project**	**Virus**		**Number of Samples**	**Cell Type**
PRJNA667921	SARS-CoV-2 ^1^		3	A549
PRJNA667921	SARS-CoV-2 ^1^		9	HEK293T
PRJNA615032	SARS-CoV-2 ^1^		12	NHBE
PRJNA615032	SARS-CoV-2 ^1^		3	Calu-3
PRJNA665581	SARS-CoV-2 ^2^		4	Calu-3
PRJNA661467	SARS-CoV-2 ^3^		34	Calu-3
PRJNA233943	SARS-CoV		42	MRC-5
PRJNA625518	SARS-CoV		54	Calu-3
PRJNA233943	MERS-CoV		53	MRC-5
PRJNA233944	MERS-CoV		32	Vero
PRJNA580021	MERS-CoV		6	Calu-3
PRJNA545350	MERS-CoV		16	mouse lung
PRJNA1062064	OC43		30	MRC-5
PRJNA679356	PEDV		3	Vero E6
(**b**)				
**Project**	**Location**	**Number of Samples**	**VOC**	**Reference**
PRJNA970221	Italy	29	alpha	Gatti et al. [15]
PRJNA970221	Italy	10	delta	Gatti et al. [15]
PRJNA970221	Italy	7	omicron variants	Gatti et al. [15]
PRJNA778445	Hong Kong	406	D614G	Chen et al., 2022 [16]
PRJNA1010395	Hong Kong	118	omicron BA.2	Chen et al., 2023 [17]
CRA004571	China	115	delta	Li et al. [18]

**Table 2:** Coordinates for expected deletion sizes for the N gene sgmRNA in viruses aligned to their own reference. R1 and R2 are the genomic coordinates for the start of the N gene TRS sequence.

Virus	Reference	N TRS Start	DEL Start	DEL End	Gene Start	R1	R2	TRS Sequence
OC43	AY391777.1	29,089	63	29,088	29,104	63	29,089	ATCTAAA
MERS	NC_019843.3	28,536	54	28,535	28,566	54	28,536	TTGATTTTAACGAA
SARS-CoV	NC_004718.3	28,103	64	28,102	28,120	64	28,103	TAAACGAAC
SARS-CoV-2	NC_045512.2	28,255	65	28,254	28,274	65	28,255	TCTAAACGAAC
PEDV	NC_003436.1	26,359	66	26,358	26,374	66	26,359	CTAAAC

**Table 3:** Nucleotide and amino acid sequences starting from TIS-L containing junctions of interest. In the Sequence column the most common sequence is shown with the junction indicated by a pipe symbol; the CTG codon for TIS-L is shown in red and the ATG start codon of the N gene in blue. An asterisk indicates a stop codon. For size ***S***, an underlined green A before the TRS-L codon indicates the first codon position of the reading frame nearby. In the Protein column, the methionine from the TRS-L ATG codon is shown in red if the reading frame is maintained.

Deletion Size	Sequence	Resulting Peptide
SARS-CoV-2		
***S***	CTGTTCTCTAAAC|GAACAAACTAAAATGTCT	LFSKRTN*NV
***S+1***	CTGTTCTCTAA|CGAACAAACTAAAATGTCT	LFSNEQTKMS
***S+4***	CTGTTCTCTAA|ACAAACTAAAATGTCT	LFSKQTKMS
SARS-CoV		
***S***	CTGTTCTCTAAAC|GAACAAATTAAAATGTCT	LFSKRTN*NV
***S+1***	CTGTTCTCTAA|CGAACAAATTAAAATGTCT	LFSNEQIKMS
***S+4***	CTGTTCTCTAA|ACAAATTAAAATGTCT	LFSKQIKMS

**Table 4:** The effect of the 1 bp deletion at 28271 nt on nucleotide and amino acid sequences starting from TIS-L for the SARS-CoV-2 deletions in Table 3. An asterisk indicates a stop codon. Underscores in nucleotide sequences indicate the position of the 1 bp deletion.

Deletion Size	Sequence	Resulting Peptide
SARS-CoV-2		
***S***	CTGTTCTCTAAAC|GAACAAACTAA_ATGTCT	LFSKRTN*MS
***S+1***	CTGTTCTCTAA|CGAACAAACTAA_ATGTCT	LFSNEQTKC
***S+4***	CTGTTCTCTAA|ACAAACTAA_ATGTCT	LFSKQTKC

## Data Availability

All results are available in the paper and Supplementary Materials.

## Supplementary Materials

The following supporting information can be downloaded at: https://www.mdpi.com/article/10.3390/v17010036/s1, Figure S1. Repeated sequences on either side of size S junction in OC43 are underlined. All possible alignments are shown for that preserve the sequence of the read. For a repeat of length L bp there are L+1 possible alignments. In this example the repeat is of length 7 and there are 8 possible alignments. Table S1: Percentage of samples containing deletions of sizes in the S-1 to S+4 range in SARS-CoV-2 cell line sample groups.; Table S2. Percentage of samples containing deletions of sizes in the S-1 to S+4 range in SARS-CoV-2 patient sample groups. Table S3. Percentage of samples containing deletions of sizes in the S-1 to S+4 range in SARS-CoV sample groups. Table S4. Percentage of samples containing deletions of sizes in the S-1 to S+2 range in MERS-CoV sample groups. S+3 and S+4 were not present in significant amounts. Table S5. Percentage of samples containing deletions of sizes in the S-1 to S+4 range in an OC43 sample group. Table S6. Sequences of Interest for SARS-CoV-2, SARS-CoV, MERS-CoV, OC43, and PEDV. Sequences of interest for OC43 adjusted to account for the SNV T59C. Table S7. Sequences of interest for SARS-CoV-2 RPF.

## References

[1] Grigoriev A. (2004). Mutational patterns correlate with genome organization in SARS and other coronaviruses. Trends Genet..

[2] Kim D., Lee J.-Y., Yang J.-S., Kim J.W., Kim V.N., Chang H. (2020). The architecture of SARS-CoV-2 transcriptome. Cell.

[3] El-Maradny Y.A., Badawy M.A., Mohamed K.I., Ragab R.F., Moharm H.M., Abdallah N.A., Elgammal E.M., Rubio-Casillas A., Uversky V.N., Redwan E.M. (2024). Unraveling the role of the nucleocapsid protein in SARS-CoV-2 pathogenesis: From viral life cycle to vaccine development. Int. J. Biol. Macromol..

[4] Kim D., Kim S., Park J., Chang H.R., Chang J., Ahn J., Park H., Park J., Son N., Kang G. (2021). A high-resolution temporal atlas of the SARS-CoV-2 translatome and transcriptome. Nat. Commun..

[5] Kearse M.G., Wilusz J.E. (2017). Non-AUG translation: A new start for protein synthesis in eukaryotes. Genes Dev..

[6] Finkel Y., Mizrahi O., Nachshon A., Weingarten-Gabbay S., Morgenstern D., Yahalom-Ronen Y., Tamir H., Achdout H., Stein D., Israeli O. (2021). The coding capacity of SARS-CoV-2. Nature.

[7] Martin M. (2011). Cutadapt removes adapter sequences from high-throughput sequencing reads. EMBnet. J..

[8] Li H., Durbin R. (2009). Fast and accurate short read alignment with Burrows–Wheeler transform. Bioinformatics.

[9] Li H., Handsaker B., Wysoker A., Fennell T., Ruan J., Homer N., Marth G., Abecasis G., Durbin R., Subgroup G.P.D.P. (2009). The sequence alignment/map format and SAMtools. Bioinformatics.

[10] Orgera J., Kelley J.J., Bar O., Vaidhyanathan S., Grigoriev A. (2022). SARSNTdb database: Factors affecting SARS-CoV-2 sequence conservation. Front. Virol..

[11] Condé L., Allatif O., Ohlmann T., de Breyne S. (2022). Translation of SARS-CoV-2 gRNA is extremely efficient and competitive despite a high degree of secondary structures and the presence of an uORF. Viruses.

[12] Yang D., Leibowitz J.L. (2015). The structure and functions of coronavirus genomic 3′ and 5′ ends. Virus Res..

[13] Mackeown M., Kung Y.-A., Davila-Calderon J., Ford W.P., Luo L., Henry B., Li M.-L., Brewer G., Shih S.-R., Tolbert B.S. (2023). The 5′ UTR of HCoV-OC43 adopts a topologically constrained structure to intrinsically repress translation. J. Biol. Chem..

[14] Madhugiri R., Fricke M., Marz M., Ziebuhr J. (2014). RNA structure analysis of alphacoronavirus terminal genome regions. Virus Res..

[15] Gatti G., Brandolini M., Mancini A., Taddei F., Zannoli S., Dirani G., Manera M., Arfilli V., Denicolò A., Marzucco A. (2023). Genomic and Temporal Analysis of Deletions Correlated to qRT-PCR Dropout in N Gene in Alpha, Delta and Omicron Variants. Viruses.

[16] Chen Z., Ng R.W.Y., Lui G., Ling L., Chow C., Yeung A.C.M., Boon S.S., Wang M.H., Chan K.C.C., Chan R.W.Y. (2022). Profiling of SARS-CoV-2 subgenomic RNAs in clinical specimens. Microbiol. Spectr..

[17] Chen Z., Ng R.W.Y., Lui G., Ling L., Leung A.S., Chow C., Boon S.S., Ho W.C., Wang M.H., Chan R.W.Y. (2024). Quantitative and qualitative subgenomic RNA profiles of SARS-CoV-2 in respiratory samples: A comparison between Omicron BA. 2 and non-VOC-D614G. Virol. Sin..

[18] Li B., Deng A., Li K., Hu Y., Li Z., Shi Y., Xiong Q., Liu Z., Guo Q., Zou L. (2022). Viral infection and transmission in a large, well-traced outbreak caused by the SARS-CoV-2 Delta variant. Nat. Commun..

[19] Irigoyen N., Firth A.E., Jones J.D., Chung B.Y.-W., Siddell S.G., Brierley I. (2016). High-resolution analysis of coronavirus gene expression by RNA sequencing and ribosome profiling. PLoS Pathog..

[20] Dinan A.M., Keep S., Bickerton E., Britton P., Firth A.E., Brierley I. (2019). Comparative analysis of gene expression in virulent and attenuated strains of infectious bronchitis virus at subcodon resolution. J. Virol..

[21] Graber T.E., Mercier É., Bhatnagar K., Fuzzen M., D’Aoust P.M., Hoang H.-D., Tian X., Towhid S.T., Plaza-Diaz J., Eid W. (2021). Near real-time determination of B. 1.1. 7 in proportion to total SARS-CoV-2 viral load in wastewater using an allele-specific primer extension PCR strategy. Water Res..

[22] Rosato A.E., Msiha E., Weng B., Mesisca M., Gnass R., Gnass S., Bol C., Tabuenca A., Rosato R.R. (2022). Rapid detection of the widely circulating B. 1.617. 2 (Delta) SARS-CoV-2 variant. Pathology.

[23] Pipek O.A., Medgyes-Horváth A., Stéger J., Papp K., Visontai D., Koopmans M., Nieuwenhuijse D., Oude Munnink B.B., Csabai I., VEO Technical Working Group (2024). Systematic detection of co-infection and intra-host recombination in more than 2 million global SARS-CoV-2 samples. Nat. Commun..

[24] Thorne L.G., Bouhaddou M., Reuschl A.-K., Zuliani-Alvarez L., Polacco B., Pelin A., Batra J., Whelan M.V., Hosmillo M., Fossati A. (2022). Evolution of enhanced innate immune evasion by SARS-CoV-2. Nature.

[25] Lee K.S., Wong T.Y., Russ B.P., Horspool A.M., Miller O.A., Rader N.A., Givi J.P., Winters M.T., Wong Z.Y., Cyphert H.A. (2022). SARS-CoV-2 Delta variant induces enhanced pathology and inflammatory responses in K18-hACE2 mice. PLoS ONE.

[26] Mulroney T.E., Pöyry T., Yam-Puc J.C., Rust M., Harvey R.F., Kalmar L., Horner E., Booth L., Ferreira A.P., Stoneley M. (2024). N(1)-methylpseudouridylation of mRNA causes +1 ribosomal frameshifting. Nature.

[27] Grossoehme N.E., Li L., Keane S.C., Liu P., Dann III C.E., Leibowitz J.L., Giedroc D.P. (2009). Coronavirus N protein N-terminal domain (NTD) specifically binds the transcriptional regulatory sequence (TRS) and melts TRS-cTRS RNA duplexes. J. Mol. Biol..

[28] Mears H.V., Young G.R., Sanderson T., Harvey R., Crawford M., Snell D.M., Fowler A.S., Hussain S., Nicod J., Peacock T.P. (2022). Emergence of new subgenomic mRNAs in SARS-CoV-2. BioRxiv.

[29] Jungreis I., Sealfon R., Kellis M. (2021). SARS-CoV-2 gene content and COVID-19 mutation impact by comparing 44 Sarbecovirus genomes. Nat. Commun..

[30] Kozak M. (1995). Adherence to the first-AUG rule when a second AUG codon follows closely upon the first. Proc. Natl. Acad. Sci. USA.

