# Supplementary material for: Patterns of Isoform Variation for N Gene Subgenomic mRNAs in Betacoronavirus Transcriptomes
Source: Viruses. 2024 Dec 30;17(1):36. doi: 10.3390/v17010036 (PMC11769239; doi:10.3390/v17010036)
Supplement: Supplementary file 1 [file viruses-17-00036-s001.zip › supplementary_tables.pdf]

Supplementary Information

				PRJNA665581	PRJNA615032	PRJNA615032	PRJNA667921	PRJNA667921
Size	Start	End	Length	Calu-3	NHBE	Calu-3	A549	HEK293T
28189	65	28253	S-1	70	0	100	100	100
28189	71	28259	S-1	20	0	0	33	33
28190	70	28259	S	100	100	100	100	100
28191	70	28260	S+1	30	0	100	100	100
28192	65	28256	S+2	0	0	100	100	100
28192	69	28260	S+2	0	0	33	0	22
28194	72	28265	S+4	90	8	100	100	100

Table S1. Percentage of samples containing deletions of sizes in the ***S-1*** to ***S+4*** range in SARS-CoV-2 cell line sample groups.

				PRJNA1010395	PRJNA778445	PRJNA970221	PRJNA970221	PRJNA970221	CRA004571
Size	Start	End	Length	omicron	D614G	alpha	delta	omicron	delta
28189	65	28253	S-1	28	24	60	0	57	0
28189	71	28259	S-1	0	1	7	9	0	1
28190	70	28259	S	99	93	93	91	100	96
28191	70	28260	S+1	15	28	0	36	0	3
28192	65	28256	S+2	0	8	0	9	0	0
28192	69	28260	S+2	5	2	0	0	0	0
28194	72	28265	S+4	62	44	83	91	100	39

Table S2. Percentage of samples containing deletions of sizes in the ***S-1*** to ***S+4*** range in SARS-CoV-2 patient sample groups.

				PRJNA233943	PRJNA625518	PRJNA625518	PRJNA625518
Size	Start	End	Length	MRC5	Calu-3 Single Poly A	Calu-3 Paired Poly A	Calu-3 Paired total
28038	68	28105	S-1	21	88	75	100
28039	69	28107	S	86	100	100	100
28040	67	28106	S+1	17	62	25	100
28041	62	28102	S+2	5	12	0	100
28043	69	28111	S+4	14	12	0	100

Table S3. Percentage of samples containing deletions of sizes in the S-1 to S+4 range in SARS-CoV sample groups.

				PRJNA233943	PRJNA233944	PRJNA580021	PRJNA545350
Size	Start	End	Length	MRC5	Vero	Calu-3	mouse lungs
28481	59	28539	S-1	70	50	100	6
28482	59	28540	S	100	100	100	100
28483	59	28541	S+1	68	53	100	6
28484	59	28542	S+2	58	50	100	12

Table S4. Percentage of samples containing deletions of sizes in the S-1 to S+2 range in MERS-CoV sample groups. S+3 and S+4 were not present in significant amounts.

Size	Start	End	Length	GSE252692
29025	63	29087	S-1	38
29025	64	29088	S-1	50
29026	70	29095	S	98
29027	69	29095	S+1	58
29027	70	29096	S+1	58
29028	64	29091	S+2	38
29028	69	29096	S+2	48
29028	70	29097	S+2	52
29030	64	29093	S+4	22

Table S5. Percentage of samples containing deletions of sizes in the S-1 to S+4 range in an OC43 sample group.

Size	Start	End	Sequence of Interest
SARS-CoV-2			
28189	65	28253	CTTGTAGATCTGTTCATCTAAACGAACAAACTAAAATG
28189	71	28259	CTGTTCTCTAAAACGAACAAACTAAAATG
28190	70	28259	CTGTTCTCTAAACGAACAAACTAAAATG
28191	70	28260	CTGTTCTCTAACGAACAAACTAAAATG
28192	65	28256	CTTGTAGATCTGTTCTAAACGAACAAACTAAAATG
28192	69	28260	CTGTTCTCTACGAACAAACTAAAATG
28194	72	28265	CTGTTCTCTAAACAAACTAAAATG
SARS-CoV			
28038	68	28105	CTGTTCTCTAAAACGAACAAATTAAAATG
28039	69	28107	CTGTTCTCTAAACGAACAAATTAAAATG
28040	67	28106	CTGTTCTCTAACGAACAAATTAAAATG
28041	62	28102	CTTGTAGATCTGTTCTAAACGAACAAATTAAAATG
28042	70	28111	CTGTTCTCTAAACGAAATTAAAATG
MERS-CoV			
28481	59	28539	TTGCAGAACTTTGATTTTTAACGAATCTCAATTTCATTGTTATG
28482	59	28540	TTGCAGAACTTTGATTTTAACGAATCTCAATTTCATTGTTATG
28483	59	28541	TTGCAGAACTTTGATTTAACGAATCTCAATTTCATTGTTATG
28484	59	28542	TTGCAGAACTTTGATTAACGAATCTCAATTTCATTGTTATG
OC43			
29025	63	29087	TAGATCTTTTCGTATATCTAAATTTTAAGGATG
29025	64	29088	TAGATCTTTTCGTAAATCTAAATTTTAAGGATG
29026	70	29095	TCGTAATCTAAATTTTAAGGATG
29027	69	29095	TCGTAATCTAATTTTAAGGATG
29027	70	29096	TCGTAATCTAAATTTAAGGATG
29028	64	29091	TAGATCTTTTCGTAATAAATTTTAAGGATG
29028	69	29096	TCGTAATCTAATTTAAGGATG
29028	70	29097	TCGTAATCTAAATTAAGGATG

29030	64	29093	TAGATCTTTTCGTAAAATTTTAAGGATG
PEDV			
26292	63	26354	TTGTCTACTCAATTTAGTCTAAACAGAAACTTTATG
26293	66	26358	TTGTCTACTCAATTCAACTAAACAGAAACTTTATG
26294	65	26358	TTGTCTACTCAATTCACTAAACAGAAACTTTATG
26294	68	26361	TTGTCTACTCAATTCAACTAACAGAAACTTTATG
26294	74	26367	TTGTCTACTCAATTCAACTAAACAGAACTTTATG
26295	72	26366	TTGTCTACTCAATTCAACTAAACAAACTTTATG

Table S6. Sequences of Interest for SARS-CoV-2, SARS-CoV, MERS-CoV, OC43, and PEDV. Sequences of interest for OC43 adjusted to account for the SNV T59C.

Size	Start	End	Sequence of Interest
28189	65	28253	CTTGTAGATCTGTTCATCTAAA
28189	71	28259	CTGTTCTCTAAAACGAACAAACT
28190	70	28259	CTGTTCTCTAAACGAACAAACT
28191	70	28260	CTGTTCTCTAACGAAC
28192	65	28256	CTTGTAGATCTGTTCTAAA
28192	69	28260	CTGTTCTCTACGAACAAACT
28194	72	28265	CTGTTCTCTAAACAAACT

Table S7. Sequences of interest for SARS-CoV-2 RPF.

```
  60        70                        29080     28090     28100
   |         |                            |         |         |
TTGTAATCTAAACTTTATAA     TGTTGAGAAATAATATCTAAATTTTAAGGATG
TTGTAATCTAAACTTTATAA     TGTTGAGAAATAATATCTAAATTTTAAGGATG
TTGTAATCTAAACTTTATAA     TGTTGAGAAATAATATCTAAATTTTAAGGATG
TTGTAATCTAAACTTTATAA     TGTTGAGAAATAATATCTAAATTTTAAGGATG
TTGTAATCTAAACTTTATAA     TGTTGAGAAATAATATCTAAATTTTAAGGATG
TTGTAATCTAAACTTTATAA     TGTTGAGAAATAATATCTAAATTTTAAGGATG
TTGTAATCTAAACTTTATAA     TGTTGAGAAATAATATCTAAATTTTAAGGATG
TTGTAATCTAAACTTTATAA     TGTTGAGAAATAATATCTAAATTTTAAGGATG
```

Figure S1. Repeated sequences on either side of size S junction in OC43 are underlined. All possible alignments are shown for that preserve the sequence of the read. For a repeat of length L bp there are L+1 possible alignments. In this example the repeat is of length 7 and there are 8 possible alignments.
